# The Prevention of Cross-Infection

**Published:** 1912-07-27

**Authors:** B. A. I. Peters

**Affiliations:** Resident Medical Officer, Ham Green Hospital, Bristol, and Lecturer on Infectious Diseases, University of Bristol.


### THE PREVENTION OF CROSS-INFECTION.

### The "Barrier" System at Ham Green Fever Hospital, Bristol.

By B. A. I. PETERS, M.B., B.C., D.P.H. Cantab., Resident Medical Officer, Ham Green Hospital, Bristol, and Lecturer on Infectious Diseases, University of Bristol.

It has long been urged by the opponents of isolation hospitals that patients undergoing treatment in them frequently contract a second disease in addition to the one for which they were admitted. This has undoubtedly been so in many cases, but the same risk is always present in any institution in which children are received for treatment. The spread of infection from one ward to another in a fever hospital has always been a rare occurrence, and is practically negligible with any reasonable precautions.

The danger arises in three ways: (1) In admitting a patient to a ward reserved for one disease, owing to a diagnostic error, when he is suffering from another disease; (2) the patient may be suffering from two infectious diseases at once; (3) he may be incubating a second disease.

The first two factors may be eliminated by isolating all doubtful and mixed cases in separate wards. This means a very heavy outlay for buildings, and a staff utterly disproportionate to the number of patients.

While the air was regarded as the chief vehicle for transference of infection, the only solution of the third difficulty appeared to be by placing each patient in a separate cubicle, because, in spite of the speedy removal of a patient developing a second disease to an isolation ward, a crop of secondary cases almost always followed, as the eruptive fevers are undoubtedly highly infectious from a very early stage.

By applying principles of modern aseptic surgical practice to every case, cross-infection may be greatly reduced without requiring an excessive number of isolation wards. The importance of "medical asepsis" was apparently first recognised in some of the French hospitals, notably the Pasteur Hospital.

The methods we have adopted for the past fifteen months at the Ham Green Hospital, Bristol, are practically an extension of the "barrier" system to every patient. Each patient on admission is provided with a separate face towel, bath towel, face flannel, aluminium comb, fine teeth comb, throat syringe, and throat brush (if necessary). These are kept exclusively for each one's use in a locker beside the bed. All eating utensils are boiled immediately after use in a large fish kettle on the ward kitchen fire. This homely but effective steriliser is used owing to the fact that we have no gas laid on to the hospital, and large electric sterilisers for this purpose use too much current.

The nursing staff when performing the patient's toilet, especially if it involves any contact with the secretions of the mouth or nose, wear rubber gloves, mounted in canvas. Before passing on to another patient the gloves are washed (without removal) in 1-500 perchloride of mercury solution. As their surface is smooth, and offers little lodgment for organisms, I consider the risk of transferring a living organism after this method of disinfection as slight. I regard the use of gloves as essential for two reasons: (1) No skin would stand the amount of constant washing and disinfection that this method entails; (2) the smooth surface of a glove is much more easily cleaned than a bare hand. After a casual handling of a patient at other times the bare hands of the nurse are carefully washed and dried. The medical officer observes the same precautions after touching a patient. In the acute wards the patients are kept in bed for at least a fortnight. By the end of this time any secondary infection they may be incubating will have developed, while in a doubtful case the diagnosis will probably have been confirmed or negatived. While this system has been in vogue (fifteen months), 1,080 patients have passed through the hospital.

In the main scarlet fever wards the following diseases have been introduced:—German measles, eleven times; whooping-cough, ten times; chicken-pox, three times; measles, three times; erysipelas, twice.

German measles spread to six other patients under circumstances which were an interesting vindication of the principle. A patient suffering from scarlet fever developed German measles a week after admission. No secondary cases arose among the other patients, but the nurse who attended this patient developed the disease within sixteen days. She was not noticed to be ill until she had washed all the patients (twelve in number) on that side of the ward, as the rash was coming out on her. After a lapse of a further sixteen days six of these patients developed the disease—the same patients who had previously been exposed without physical contact and had escaped. No secondary cases followed the invasion of whooping-cough, measles, or erysipelas.

Two secondary cases of chicken-pox followed as a result of three invasions—a result which, while disappointing, is much better than previous to the introduction of aseptic methods, when one used to find that few of those not previously protected by an attack eventually escaped. It is of interest to note that no tertiary cases of this complaint developed. If this disease breaks out in an acute ward no transference of patients to a convalescent ward is permitted. The patients, if due for getting up, are allowed up until the thirteenth day after the primary case. They are then put back to bed for five days. Any secondary cases developing are thus kept out of contact with their fellows.

It is our practice to take cultures from the noses and throats of all scarlet fever patients soon after admission to determine the presence of diphtheria carriers. Thirty-seven carriers were thus discovered, of which the majority showed no clinical evidence of diphtheria. No extension of this disease occurred.

In the diphtheria wards eight patients were found to have scarlet fever in addition to diphtheria. Measles was introduced three times and chicken-pox twice. Two secondary cases followed the invasion by chicken-pox. No secondary cases followed the other diseases.

When a definite diagnosis of a secondary disease is made (with the exception noted below) the patient is removed to a side ward opening off the main ward as an additional precaution, but is attended by the same nurses. Diphtheria carriers discovered in the scarlet fever wards are kept in the main ward. When convalescent these patients are separated from the others by a tape stretched across the main ward, and are allowed out for exercise at a different time.

All doubtful cases of scarlet fever or diphtheria are treated on the same lines as these carriers in the main ward, and in no case has cross-infection occurred.

By the use of these methods I find it rarely necessary to use the isolation block. This effects a considerable economy in staff, as such wards usually require as many nurses as patients. Dr. Rundle's * results at Fazakerly seem to show that it is not necessary to use even a side ward for doubly infected cases, but it seems advisable to make the margin of safety as wide as possible. In no case has infection spread from the side wards to the main wards.

Other infectious conditions which have been treated in the main wards without spread on several occasions are ringworm, scabies, impetigo, and vaginitis.

Another result which follows as a logical conclusion is the diminution of infectious sickness amongst the staff. For the years 1904-10 the average number of staff contracting either scarlet fever or diphtheria was nine. During the past fifteen months only four developed either disease (one scarlet fever and three diphtheria).

As a result of strict asepsis, secondary rhinitis after scarlet fever is unknown, although the primary cases remain in the main wards.

The result is that many patients are ready for discharge within a calendar month, while the return-case rate for the period under consideration has been only 0.7 per cent. (four cases from 573 discharges). The patients are discharged before desquamation is complete. I attribute this low rate to the fact that patients do not become re-infected from each other.

The one disadvantage of the system is that no books or toys can be given from a common stock to the patients. Any such articles sent in by the friends are destroyed when the patient leaves. The training of probationers in aseptic medical methods, once the ward sisters have grasped the principles, presents no great difficulties.

The advantages of the system appear to be: (1) That the risk of contracting a second disease in hospital is reduced to a minimum; (2) that many doubtful cases may be treated in the main wards without danger to themselves or others, thus effecting a great saving in isolation wards; (3) the hospital can be administered successfully by the use of side wards without utilising a separate isolation block; (4) the average duration of the patient's stay in hospital can be reduced without causing any increase in the return case rate.

* Proceedings Royal Society of Medicine, Epidemiological Section, April 26, 1912.

